# Comparative evaluation of antiproliferative, antiangiogenic and apoptosis inducing potential of black tea polyphenols in the hamster buccal pouch carcinogenesis model

**DOI:** 10.1186/1477-3163-6-19

**Published:** 2007-12-03

**Authors:** Paramasivame Vidjaya Letchoumy, Kurapathy Venkata Poorna Chandra Mohan, Duvuru Prathiba, Yukihiko Hara, Siddavaram Nagini

**Affiliations:** 1 Department of Biochemistry and Biotechnology, Faculty of Science, Annamalai University, Annamalainagar-608 002, Tamil Nadu, India; 2 Department of Pathology, Sri Ramachandra Medical College and Research Institute, Chennai-600 116, Tamil Nadu, India; 3 Mitsui Norin Co. Ltd., Tokyo, Japan

## Abstract

**Background:**

To evaluate the relative chemopreventive efficacy of two black tea polyphenols, Polyphenon-B [P-B] and BTF-35 on 7,12-dimethylbenz [a]anthracene (DMBA)-induced hamster buccal pouch (HBP) carcinogenesis.

**Methods:**

Hamsters were divided into 6 groups. The right buccal pouches of animals in groups 1–3 were painted with 0.5% of DMBA three times a week for 14 weeks. While hamsters in group 1 received no further treatment, animals in groups 2 and 3 received diet containing 0.05% P-B and BTF-35 respectively, four weeks before DMBA painting that was continued until the end of the experiments. Animals in groups 4 and 5 were given P-B and BTF-35 alone respectively as in groups 2 and 3. Group 6 animals served as the untreated control. All the animals were sacrificed after 18 weeks. The expression of p21, cyclin D1, glutathione S-transferase pi (GST-P), nuclear factor kappa B (NF-κB), Bcl-2, Bax, cytochrome C, caspase-3, caspase-9, poly(ADP-ribose) polymerase (PARP), cytokeratins and vascular endothelial growth factor (VEGF) was analysed by RT-PCR, immunohistochemical and Western blot analyses.

**Results:**

DMBA treated animals developed buccal pouch carcinomas that displayed increased expression of p21, cyclin D1, GST-P, NF-κB, cytokeratins, VEGF and Bcl-2 with decreased expression of Bax, cytochrome C, caspase-3, caspase-9, and PARP. Dietary administration of both P-B and BTF-35 reduced the incidence of DMBA-induced HBP carcinomas by modulating markers of cell proliferation, cell survival, tumour infiltration, angiogenesis, and apoptosis.

**Conclusion:**

The results of the present study provide a mechanistic basis for the chemopreventive potential of black tea polyphenols. The greater efficacy of BTF-35 in inhibiting HBP carcinogenesis and modulating multiple molecular targets may have a potential role in the prevention of oral cancer.

## Background

Oral squamous cell carcinoma (OSCC), the fifth most common cancer worldwide is a major cause of morbidity and mortality in India [1]. Recent efforts to control the incidence of OSCC have focused on developing effective chemoprevention strategies. However, it is important to establish chemoprevention in an experimental animal tumour model that mimics specific characteristics of human OSCC.

The hamster buccal pouch (HBP) carcinogenesis model is the most well characterized animal system for the investigation of oral cancer development and intervention by chemopreventive agents. The buccal pouch of the Syrian hamster serves as an excellent target organ for chemointervention because of easy accessibility for examination and follow-up of lesions. Squamous cell carcinoma (SCC) induced by 7,12-dimethylbenz [a]anthracene (DMBA) in the HBP reiterates many of the features observed in human OSCC. Topical application of DMBA to the HBP induces SCC preceded by hyperplasia, papilloma and dysplasia similar to human OSCC [2]. The major risk factors associated with human oral cancer such as tobacco, betel quid and alcohol also promote HBP carcinogenesis [3,4]. The development of both OSCC and HBP carcinomas is associated with sustained genetic mutations that lead to excessive cell proliferation, prolonged cell survival, dysregulation of cellular differentiation and evasion of apoptosis [2]. The similarities between human OSCC and HBP carcinomas provide a rationale for analyzing the effect of putative chemopreventive agents in the HBP model. We have used this model extensively in this laboratory to test the chemopreventive efficacy of several medicinal plants, phytochemicals and dietary agents [5,6].

Chemoprevention by dietary agents has evolved as an effective strategy to control the incidence of oral cancer. Epidemiological studies have demonstrated a positive correlation between increased consumption of vegetables, fruits and beverages with reduced risk of cancer [7,8]. Recently, a great deal of attention has been focused on tea made from the leaves of *Camellia sinensis*. About 3 billion kilograms of tea are produced and consumed annually, of which black tea accounts for nearly 80 per cent. Several studies have demonstrated the protective effects of black tea against chronic diseases such as cardiovascular disease and cancer [8,9]. The predominant consumption of black tea has intensified global interest to evaluate the protective effect of black tea and its constituents against carcinogenesis.

Black tea is known to exhibit a wide spectrum of pharmacological effects including antioxidant, antiproliferative, anti-inflammatory and immunomodulatory properties [8]. In previous reports from this laboratory, we documented the chemopreventive potential of a mixture of black tea polyphenols (Polyphenon-B; [P-B]) both alone and in combination with bovine lactoferrin in the HBP carcinogenesis model [10]. We have also demonstrated the inhibitory effects of P-B on rat mammary and stomach carcinogenesis models [11,12]. The present study was designed to compare the chemopreventive potential of polyphenon- B with BTF-35, a black tea extract enriched with theaflavins and catechins, in the HBP model. Since no single marker can fully elucidate the mechanism underlying chemoprevention, we analysed the expression of a panel of markers that are reliable indicators of cell proliferation and survival (p21, cyclin D1, glutathione S-transferase pi; GST-P, and nuclear factor kappa B; NF-κB), apoptosis (Bcl-2, Bax, cytochrome C, caspase-3, caspase-9, and poly(ADP-ribose) polymerase; PARP), as well as tumour invasion and angiogenesis (cytokeratins and vascular endothelial growth factor, VEGF) by RT-PCR, immunohistochemical, and Western blot analyses. In addition, the activity of caspase-3 was assayed by a colorimetric method. Our results indicate that BTF-35 is a more potent inhibitor of HBP carcinogenesis based on suppression of HBP carcinomas and modulation of markers of cell proliferation, cell survival, tumour infiltration, angiogenesis and apoptosis.

## Methods

### Chemicals

DMBA was purchased from Sigma Chemical Company, St. Louis, MO, USA. P-B and BTF-35 were kindly provided by Mitsui Norin Co., Ltd., Tokyo, Japan. The composition of P-B and BTF-35 are given in Table 1. All other reagents used were of analytical grade.

**Table 1:** Composition of P-B and BTF-35

**Polyphenolic constituent**	**P-B (%w/w)**	**BTF-35 (%w/w)**
Epigallocatechin (EGC)	-	0.1
Epicatechin (EC)	0.4	0.2
Epigallocatechin gallate (EGCG)	1.4	2.6
Epicatechin gallate (ECG)	0.1	2.1
Gallocatechin gallate (GCG)	0.2	0.3
Catechin gallate (CG)	-	0.1
Catechin (C)	-	0.1
Free theaflavin	0.32	7.1
Theaflavinmonogallate-A	0.14	8.3
Theaflavinmonogallate-B	0.15	2.6
Theaflavindigallate	0.24	9.8
Tannin	35.6	-
Caffeine	4.9	0.5

### Animals

The experiment was carried out with male Syrian hamsters aged 6–10 weeks weighing between 90–110 g obtained from the Central Animal House, Annamalai University, India. The animals were housed six to a polypropylene cage and provided food and water *ad libitum*. The animals were maintained in a controlled environment under standard conditions of temperature and humidity with an alternating 12-hours light/dark cycle. The animals were maintained in accordance with the guidelines of the Indian Council of Medical Research and approved by the ethical committee, Annamalai University. Experimental diet was prepared everyday by mixing P-B and BTF-35 to preweighed standard pellet diet (Mysore Snack Feed, Mysore, India). The diet was replenished everyday and the food consumption was recorded.

### Treatment schedule

The hamsters were randomized into experimental and control groups and divided into 6 groups of 6 animals each. At 10 weeks of age, the hamsters in groups 1 to 3 were painted with a 0.5% solution of DMBA in liquid paraffin on the right buccal pouches using a number 4 brush three times a week for 14 weeks. Each application leaves approximately 0.4 mg DMBA [2]. Hamsters in group 1 received no further treatment. Animals in groups 2 and 3 received diet containing P-B and BTF-35 respectively four weeks before DMBA painting when they were 6 weeks of age and continued until the final exposure to carcinogen. Animals in groups 4 and 5 were given P-B and BTF-35 alone respectively as in groups 2 and 3. Group 6 animals received basal diet and served as control. The dose for black tea polyphenols used in the present study corresponds to the daily intake of four cups of tea (30–40 mg of tea polyphenols per kilogram body weight by humans) [13]. The experiment was terminated at the end of 18 weeks and all animals were sacrificed by cervical dislocation after an overnight fast. Before an animal was killed, the right buccal pouch was grossly inspected to evaluate premalignant lesions or tumour development and photographed. The tumour burden was calculated by multiplying the mean tumour volume (4/3 πr^3^) (r = 1/2 tumour diameter in mm) with the mean number of tumours. The buccal pouch tissues were subdivided and variously processed for distribution to each experiment. A portion of the tissue was immediately frozen in liquid nitrogen for subsequent RNA extraction and another portion was processed using lysis buffer for Western blot analysis. The remaining tissues were fixed in 10% formalin, embedded in paraffin, sectioned and mounted on polylysine-coated glass slides. One section from each specimen was stained with haematoxylin and eosin. The other sections were used for immunohistochemical staining.

### Immunohistochemistry

The tissue sections on glass slides were deparaffinised by heat at 60°C for 30 minutes, followed by three washes in xylene. After gradual hydration through graded alcohol, the slides were incubated in citrate buffer (pH 6.0) for two cycles of 5 minutes in a microwave oven for antigen retrieval. The sections were allowed to cool for 20 minutes, rinsed with Tris-buffered saline (TBS) and treated with 3% H_2_O_2 _in distilled water for 15 minutes to inhibit endogenous peroxidase activity. Nonspecific antibody binding was reduced by incubating the sections with normal goat serum for 25 minutes. The sections were then incubated with mouse monoclonal antibodies against cytokeratin AE1/AE3, Bcl-2 (Dako, Carprinteria, CA, USA), caspase-3, cytochrome C (NeoMarkers, USA), p21, VEGF (Santa Cruz Biotechnology, CA, USA), and rabbit polyclonal antibodies against GST-P (BioGenex, San Raman, CA, USA), NF-κB and Bax (Santa Cruz Biotechnology) at room temperature for one hour. The slides were rinsed in TBS and incubated with anti-mouse and anti-rabbit biotin-labelled secondary antibody (DAKO) followed by streptavidin-biotin-peroxidase for 30 minutes each at room temperature. The immunoprecipitate was visualized by treating with 3,3'-diaminobenzidine (DAKO) and counterstaining with haematoxylin. For negative controls, the primary antibody was replaced with TBS. Positive controls for each antibody were also processed simultaneously.

Immunohistochemical staining was scored according to the number of positively stained cells per 100 counted cells. Immunoreactivity of p21, GST-P, NF-κB, Bcl-2, Bax, cytochrome C, caspase-3 and VEGF was regarded as negative (0) when there was no staining; weak (1) when the staining was focal and mildly intense; moderate (2) when between two-third of cells stained moderately; and strong (3) when the majority of cells (> two third) stained intensely. Cytokeratin expression was graded as 0 = failure to detect the keratin, I = staining confined either to the basal area or some evidence of suprabasal staining, II = positive staining throughout the basal and/or suprabasal region.

### Extraction of RNA

Total RNA from the hamster buccal pouch was extracted using trizol reagent (Sigma) [14]. The RNA concentration was determined from the optical density at a wavelength of 260 nm (by using an OD_260 _unit equivalent to 40 μg/ml of RNA). In brief, 50 mg pouch tissue was homogenized using (1 ml) trizol reagent. The homogenate was then treated with 0.2 ml of chloroform and shook vigorously. The mixture was then centrifuged at 12,000 *g *for 15 min at 4°C. To the aqueous phase, 0.5 ml of isopropanol was added, and centrifuged at 12,000 *g *for 8 minutes at 4°C. The supernatant was discarded gently and the precipitated RNA was rinsed twice with 1 ml of 75% ethanol and dried in air. The RNA was resuspended in 100 μl of diethylpyrocarbonate (Sigma) treated water at a final concentration of 1 μg/μl and stored at -80°C until further use.

### Reverse Transcriptase (RT) reaction: (cDNA synthesis)

Isolated total RNA (1 μg) was reverse-transcribed to cDNA in a reaction mixture containing 4 μl of 5× reaction buffer, 2 μl of dNTPs mixture (10 mM), 20 units of RNase inhibitor, 200 units of avian-myeloblastosis virus (AMV) reverse transcriptase and 0.5 μg of oligo(dT) primer (Promega, WI, USA) in a total volume of 20 μl. The reaction mixture was incubated at 42°C for 60 minutes and the reaction terminated by heating at 70°C for 10 minutes. The resultant cDNA was stored at -80°C until further use.

### PCR amplification

All oligonucleotide primers were purchased from Sigma Genosys, India. Details about the primers used for PCR reactions are given in table 2. The primers for VEGF amplified two of four different molecular species produced by alternative splicing of mRNA-VEGFI21 and VEGFI65 of sizes 444 bp and 576 bp, respectively.

**Table 2:** Oligonucleotide primers used for RT-PCR of cyclin D1, GST-P, Bcl2, Bax and VEGF with β-actin as an internal control

**Gene Product**	**Primers**	**Oligonucleotide sequences**	**Fragment size**
Cyclin D1	Sense Antisense	5'-CGGAGGACAACAAACAGATC-3' 5'-GGGTGTGCAAGCCAGGTCCA-3'	331 bp
GST-P	Sense Antisense	5'-TCATCTACACCAACTATGAG-3' 5'-GCCACATAGGCAG AGAGCAG-3'	226 bp
Bcl-2	Sense Antisense	5'-TGCACCTGACGCCCTTCAC-3' 5'-AGACAGCCAGG AGAAATCA AACAG-3'	293 bp
Bax	Sense Antisense	5'-ACCAAG CTGAGCGA GTGTC-3' 5'-ACAAAGATGGTCACGGTCTGCC-3'	374 bp
VEGF	Sense Antisense	5'-ATGAACTTTCTGCTGTCTTGG-3' 5'-TCACCGCCTCGGCTTGTCACA-3'	444 bp and 576 bp*
β-actin	Sense Antisense	5'-AACCGCGAGAAGATGACCCAGATCATGTTT-3' 5'-AGCAGCCGTGGCCATC TCTTGCTCGAAGTC-3'	350 bp

*The primers for VEGF detect two of four different molecular species produced by alternative splicing of mRNA-VEGFI21 and VEGFI65, with expected fragment sizes of 444 bp and 576 bp, respectively.

The PCR amplification reaction mixture (in a final volume of 25 μl) contained 1 μl of cDNA, 0.5 μl of forward primer, 0.5 μl of reverse primer and 10 μl of Hot Master Mix (2.5×) (Eppendorf, Hamburg, Germany). The PCR was carried out in a thermal cycler (Eppendorf, Hamburg, Germany). Thermocycling conditions included initial denaturation at 94°C for 5 minutes/10 minutes for VEGF (one cycle), then denaturation at 95°C for 1 minute/30 seconds for VEGF, annealing at 55°C (1 minute) for cyclin D1, GST-P, Bcl-2 and Bax, 51°C (30 seconds) for VEGF and 60°C (1 minute) for β-actin, and extension at 72°C (1 minute) for 30 cycles (40 cycles for VEGF) and a final extension at 72°C for 7 minutes. Negative controls without cDNA were also performed. Amplification products were analysed by electrophoresis in a 2% agarose gel containing ethidium bromide with 100 bp DNA ladder. The PCR products were visualized as bands with a UV-transilluminator and photographs were taken using gel documentation system (Gel Doc Mega, United Kingdom).

### SDS-PAGE and Western blot analysis

Approximately, 50 mg of each tissue sample was subjected to lysis in a sample buffer containing 62.5 mM Tris (pH 6.8), 2% SDS, 5% 2-mercaptoethanol, 10% glycerol and bromophenol blue. The protein concentration of lysates was determined by Bradford method [15]. SDS-PAGE was performed using equivalent protein extracts (55 μg) from each sample according to Laemmli [16]. The resolved proteins were electrophoretically transferred to polyvinylidene difluoride membranes (Sartorius, Germany). The membranes were incubated in 1× PBS containing 5% non-fat dry milk for 2 h to block non-specific binding sites. The blots were incubated with 1:400 dilution of anti-NF-κB, Bcl-2, Bax, caspase-3, caspase-9, PARP and VEGF (Santa Cruz Biotechnology) overnight at 4°C. The blots were washed thrice with high salt buffer (2.18 g NaH_2_PO_4, _7 g Na_2_HPO_4_, 23.37 g NaCl and 200 μl Triton ×-100 in 400 ml distilled water) followed by low salt buffer (2.18 g NaH_2_PO_4, _7.03 g Na_2_HPO_4_, 1.2 g NaCl and 200 μl Triton ×-100 in 400 ml distilled water). The blots were then incubated with 1:1000 dilution of horse radish peroxidase-conjugated secondary antibodies (Santa Cruz Biotechnology) for 45 minutes at room temperature. After extensive washes with high and low salt buffers, the immunoreactive proteins were visualized using enhanced chemiluminescence (ECL) detection reagents (Sigma). Densitometry was performed on IISP flat bed scanner and quantitated with Total Lab 1.11 software.

### Colorimetric estimation of caspase-3 activity

DEVD-specific caspase-3 activity was assayed using CASP-3-C colorimetric kit (Sigma) according to the manufacturer's instructions. Cytosolic extracts were prepared by homogenizing tissues in lysis buffer containing 50 mM HEPES (pH 7.4), 5 mM CHAPS and 5 mM DTT. The supernatant was collected as an enzyme source. The caspase-3 colorimetric assay is based on the hydrolysis of the peptide substrate acetyl-Asp-Glu-Val-Asp-nitroanilide (Ac-DEVD-pNA) by caspase-3, resulting in release of the p-nitroaniline (pNA) moiety. The concentration of the pNA released from the substrate is calculated from the absorbance values at 405 nm or from a calibration curve prepared with defined pNA solutions.

### Statistical analysis

The data are expressed as mean ± SD. Tumour incidence and grading of p21, GST-P, NF-κB, Bcl-2, Bax, cytochrome C, caspase-3, cytokeratin and VEGF were statistically compared using χ^2^-test. Statistical analysis on the data for tumour burden was carried out using Student's *t *test. The data for densitometric analysis, Bcl-2/Bax ratio and colorimetric assay of caspase-3 were analysed using Analysis of variance (ANOVA) followed by Least Significant Difference (LSD). The results were considered statistically significant if the *p *value was < 0.05.

## Results

Table 3 shows the tumour incidence, mean tumour burden and histopathological changes in experimental animals. The tumour incidence in group 1 was 100 per cent with a mean tumour burden of 127 mm^3^. Histologically, HBP tumours induced by DMBA were invasive squamous cell carcinomas with papillary projections of squamous epithelium into the connective tissues. Administration of P-B and BTF-35 effectively suppressed the development of HBP carcinomas. One of the 6 animals treated with DMBA and P-B developed SCC, while 2 animals showed mild to moderate dysplasia and remaining 3 hamsters displayed moderate hyperplasia. No tumours were observed in animals treated with DMBA and BTF-35 while 2 out of 6 animals showed moderate hyperplasia. In groups 4–6, the epithelium was normal, intact and continuous.

**Table 3:** Tumour incidence, tumour burden and histopathological changes in the buccal pouch of experimental animals (mean ± SD; n = 6)

Group	Treatment	Tumour incidence	Tumour burden^a ^mm^3^	Hyperplasia	Dysplasia	Squamous cell carcinoma
1.	DMBA	6/6	127.21 ± 87.42	+++	+++	**# **(100%)
2.	DMBA+ P-B	1/6 ^b^	8.31 ± 7.92^c^	++	+/++	**# **(16.6%)^b^
3.	DMBA+ BTF-35	0/6^d^	-	+	-	-
4	Control	0/6	-	-	-	-

+ = mild; ++ = moderate; +++ = severe; # = well differentiated; - = no change; Parentheses, percentage of lesions

a – Mean tumour burden was calculated by multiplying the mean tumour volume with the mean number of tumours (tumour volume was calculated using 4/3 πr^3^, where r = 1/2 tumour diameter in mm)

b – Significantly different from group 1 by χ^2^-test (p < 0.05)

c – Significantly different from group 1 by Student's *t *test (p < 0.001)

d – Significantly different from group 1 by χ^2^-test (p < 0.001)

**Figure 1:**
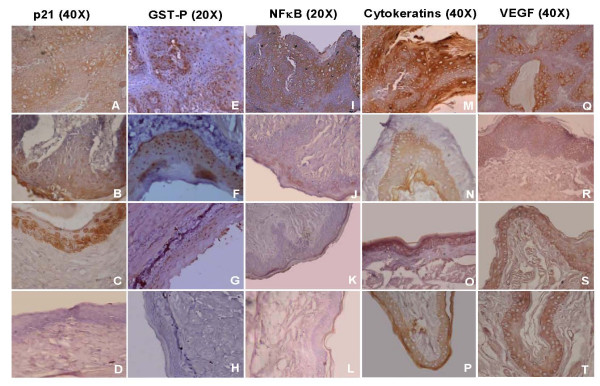
Photomicrographs of immunohistochemical staining of p21, GST-P, NF-κB, cytokeratins and VEGF in control and experimental animals. A Increased cytoplasmic expression of p21 in group 1 animals B & C Increased nuclear expression of p21 in groups 2 and 3 animals, D Normalexpression of p21 in groups 4, 5 and 6 animals, E Overexpression, ofGST-P in group 1 animals, F & G Downregulation of GST-P in groups 2 and 3 animals, H Normal expression of GST-P in groups 4, 5 and 6 animals, I Overexpression of NF-κB in group 1 animals, J & K Downregulation of NF-κB in groups 2 and 3 animals, L Normal expression of NF-κB ingroups 4, 5 and 6 animals, M, Overexpression of cytokeratins in group1 animals, N &O Downregulation of cytokeratins in groups 2 and 3 animals, P Normal expression of cytokeratins in groups 4, 5 and 6 animals, Q Overexpression of VEGF in group 1 animals, R& S Downregulation of VEGF in groups 2 and 3 animals, T Normal expression of VEGF in groups 4, 5 and 6 animals.

Figure 1 and Table 4 depict the immunohistochemical analysis of p21, GST-P, NF-κB, cytokeratins and VEGF in the hamster buccal pouch of control and experimental animals. Topical application of DMBA (group 1) significantly increased the expression of p21, GST-P, NF-κB, cytokeratins and VEGF as compared to control (group 6). In contrast to cytoplasmic staining of p21 in DMBA painted animals, the pattern of p21 staining in P-B and BTF-35 treated animals (groups 2 and 3), was predominantly in the nucleus with an occasional cell showing cytoplasmic staining. Dietary administration of P-B and BTF-35 significantly decreased the expression of GST-P, NF-κB, cytokeratins and VEGF in groups 2 and 3 animals compared to group 1. The expression of these proteins was more significantly decreased in BTF-35 treated (group 3) animals compared to P-B treated (group 2) animals. Administration of chemopreventive agents alone (groups 4 and 5) did not significantly influence protein expression compared to untreated control (group 6).

**Table 4:** Cellular localization of p21 and expression of GST-P, NF-κB, cytokeratin and VEGF in the buccal pouch of experimental and control animals (n = 6)

Group	Treatment	Cellular localization of p21 protein		p21^a^				GST-P^a^				NF-κB^a^				Cytokeratin^b^			VEGF^a^			
		
		Cytoplasm	Nucleus	0	1	2	3	0	1	2	3	0	1	2	3	I	II	III	0	1	2	3
1.	DMBA	85%^c^	<5%^c^	0	0	2	4	0^c^	0	1	5^c^	0^c^	0	1	5^c^	0	0^c^	6^c^	0	0	0	6^c^
2.	DMBA+P- B	5%^d^	73%^d^	0	0	3	3	3	1	2	0^d^	0	1	5	0^d^	0	4	2	0	3	3	0^d^
3.	DMBA+BTF-35	<5%^d^	75%^d^	0	0	2	4	4	2	0	0^d^	0	4	2	0^d^	0	5^d^	1^d^	0	5^d^	1	0^d^
4.	P-B	-	-	6	0	0	0	6	0	0	0	6	0	0	0	0	6	0	0	3	3	0
5.	BTF-35	-	-	6	0	0	0	6	0	0	0	6	0	0	0	0	6	0	0	^2^	4	0
6.	Control	-	-	6	0	0	0	6	0	0	0	6	0	0	0	0	6	0	0	2	4	0

a – Expression scored as 0- negative; 1- focal and mildly intense; 2- between one-third and two-third of cells stained moderately, and 3- majority of cells (> two third) stained intensely

b – Expression graded as 0 = failure to detect the keratin, I = staining confined either to the basal area or some evidence of suprabasal staining, II = positive staining throughout the basal and/or suprabasal region.

c – Significantly different from group 6 by χ^2^-test (p < 0.05)

d – Significantly different from group 1 by χ^2^-test (p < 0.05)

**Figure 2:**
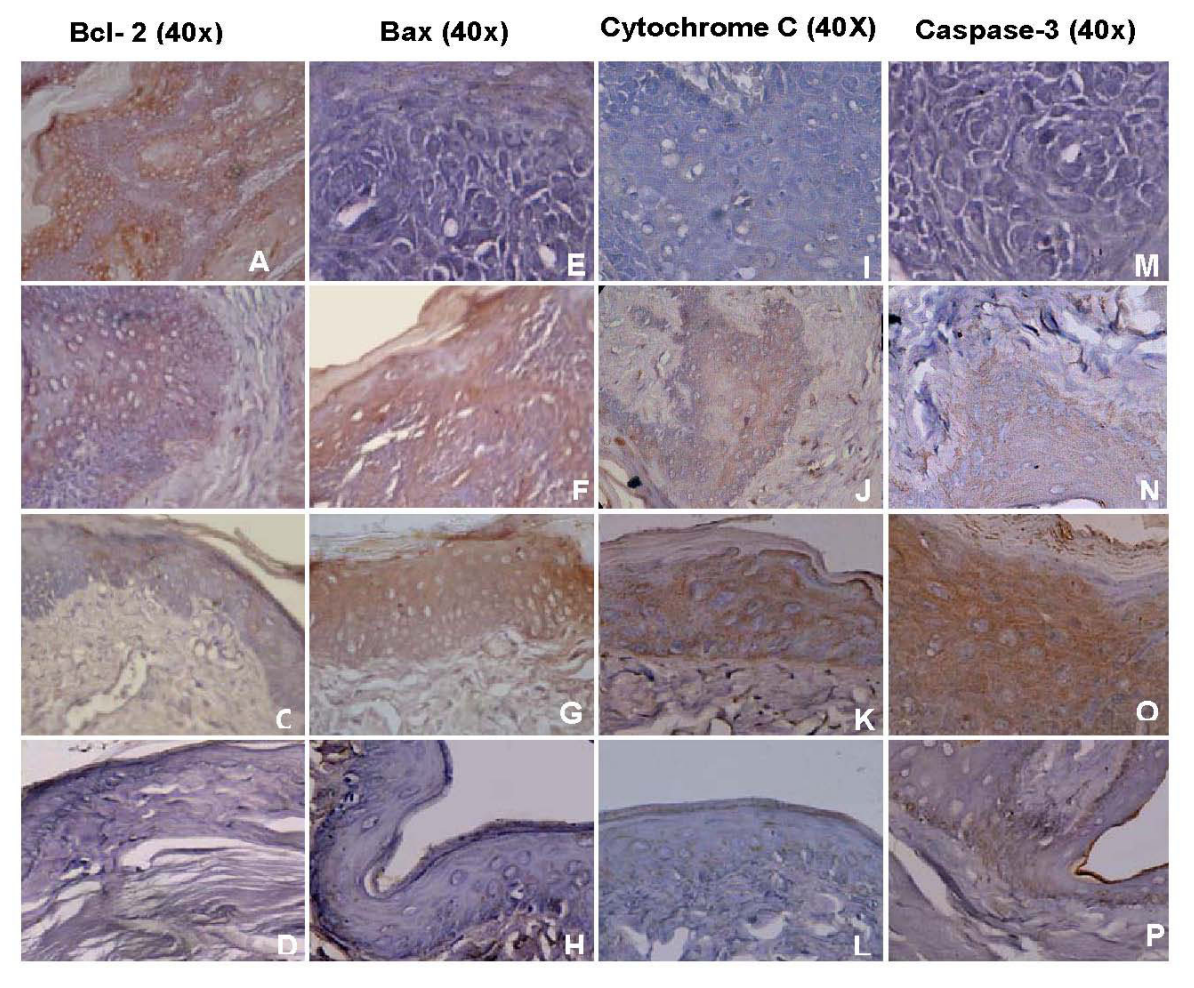
Photomicrographs of immunohistochemical staining of Bcl-2, Bax, cytochrome C and caspase 3 in control and experimental animals. A Overexpression of Bcl-2 in group 1 animals B&C Downregulation of Bcl-2 in groups 2 and 3 animals, D Normal expression of Bcl-2 in groups 4, 5 and 6 animals, E Downregulation of Bax in group 1 animals, F&G Increasedexpression of Bax in groups 2 and 3 animals, H Normal, expression of Bax in groups 4, 5 and 6 animals, I Downregulation of cytochrome C in group 1 animals, J &K Increased expression of cytochrome C in groups 2 and 3 animals, L Normal expression of cytochrome C in groups 4, 5 and 6 animals, M, Downregulation of caspase-3 in group 1 animals, N &O Increased expression of caspase-3 in groups 2 and 3 animals, P Normal expression of caspase-3 in groups 4, 5 and 6 animals.

The immunohistochemical expression of the apoptosis-associated proteins Bcl-2, Bax, cytochrome C and caspase-3 is shown in Figure 2 and Table 5. Topical application of DMBA (group 1) significantly increased the expression of Bcl-2 and decreased that of Bax, cytochrome C and caspase-3 compared to control (group 6). Administration of BTF-35 (group 3) decreased the expression of Bcl-2 and increased expression of Bax, cytochrome C and caspase-3 more significantly than P-B (group 2) compared to group 1 animals. No significant changes in the expression of proteins were observed in groups 4 and 5 as compared to control.

**Table 5:** Effect of P-B and BTF-35 on the expression of Bcl-2, Bax, cytochrome C and caspase-3 in experimental and control animals (n = 6)

Group	Treatment	Grading^a^															
		
		Bcl-2				Bax				Cytochrome C				Caspase-3			
		
		0	1	2	3	0	1	2	3	0	1	2	3	0	1	2	3
1.	DMBA	0^b^	0	1	5^b^	6^b^	0	0	0	6	0	0	0	5	1	0	0
2.	DMBA+P-B	1^c^	4	1	0^c^	0^c^	0	2	4	0^c^	1	5^c^	2	0^c^	1	3	2
3.	DMBA+BTF-35	1	5^c^	0	0	0^c^	0	1	5^c^	0^c^	0	6^c^	4	0^c^	0	2	4
4.	P-B	5	1	0	0	2	4	0	0	4	2	0	0	3	3	0	0
5.	BTF-35	6	0	0	0	1	5	0	0	3	3	0	0	2	4	0	0
6.	Control	6	0	0	0	2	4	0	0	3	3	0	0	2	4	0	0

a – Expression scored as 0- negative; 1- focal and mildly intense; 2- between one-third and two-third of cells stained moderately, and 3- majority of cells (> two third) stained intensely

b – Significantly different from group 6 by χ^2^-test (p < 0.05)

c – Significantly different from group 1 by χ^2^-test (p < 0.05)

**Figure 3:**
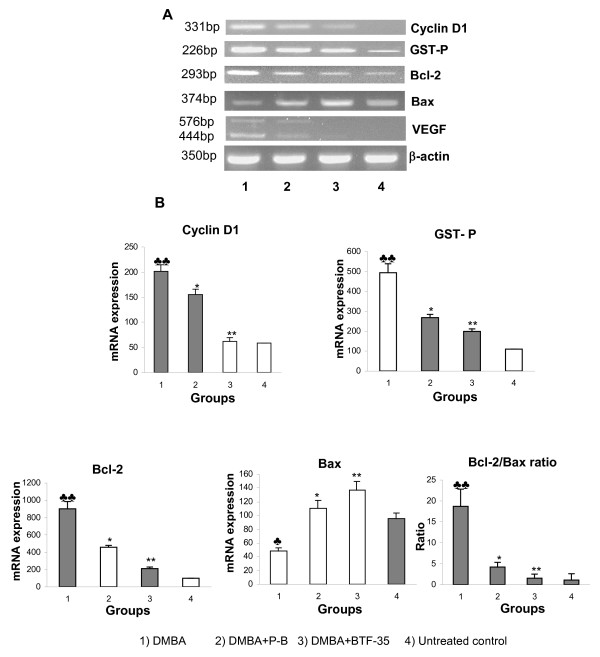
The effect of P-B and BTF-35 on the mRNA expression of cyclin D1, GST-P VEGF, Bcl-2, Bax, and Bcl-2/Bax ratio in the buccal pouch of control and experimental animals. (Mean ± SD; n = 6). (A) RT-PCR analyses of cyclin D1, GST-P, Bcl-2, Bax, and VEGF. β-actin inserted as a control (B) Densitometric analysis. ♣ Significantly different from untreated control (p < 0.001) ♣♣ Significantly different from untreated control (p < 0.0001) * Significantly different from group 1 animals (p < 0.001) ** Significantly different from group 1 animals (p < 0.0001).

Figure 3 shows the representative RT-PCR data of cyclin D1, GST-P, Bcl-2, Bax and VEGF in the buccal pouch of control and experimental animals. Quantification of each band by densitometric scanning shows significant increase in the expression of cyclin D1, GST-P, Bcl-2 and VEGF with decreased expression of Bax in DMBA painted hamsters (group 1) compared to untreated control. Although both P-B and BTF-35 significantly decreased cyclin D1, GST-P, VEGF expression and Bcl-2/Bax ratio compared to group 1 animals, BTF-35 was more effective. No significant changes in the mRNA expression of cyclin D1, GST-P, Bcl-2, Bax and VEGF were observed in animals treated with chemopreventive agents alone as compared to control.

**Figure 4:**
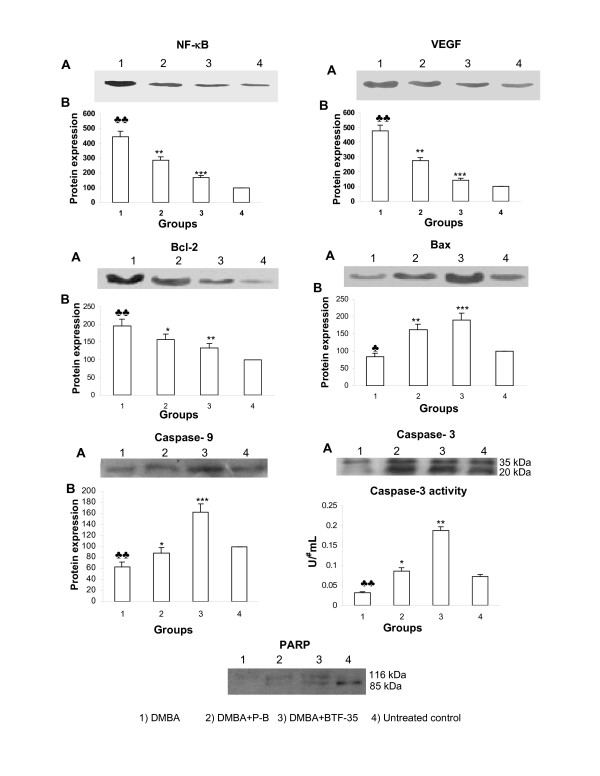
The effect of P-B and BTF-35 on NF-κB, VEGF, Bcl-2, Bax, caspase-3, caspase-9, and ARP expression and caspase-3 activity in the buccal pouch of control and experimental animals (Mean ± SD; n = 6). A) Representative immunoblots of NF-κB, VEGF, Bcl-2, Bax, caspase-3, caspase-9, and PARP (The intact caspase-3 (35 kDa) and a cleavage fragment (20 kDa) as well as the intact PARP (116 kDa) and a PARP cleavage fragment (85 kDa) are indicated). (B) Densitometric analysis. ♣ Significantly different from untreated control (p < 0.01), ♣♣ Significantly different from untreated control (p < 0.001)* Significantly different from group 1 animals (p < 0.01), ** Significantly different from group 1 animals (p < 0.001), *** Significantly different from group 1 animals (p < 0.0001)U^# ^– μmoles of pNA formed/min.

Figure 4 shows representative Western blot analysis of NF-κB, VEGF, Bcl-2, Bax, caspase-3, caspase-9, and PARP and the activity of DEVD-specific caspase-3 in the buccal pouch of control and experimental animals. The expression of NF-κB, VEGF, Bcl-2, Bax and caspase-9 was detected as bands of molecular weight, 65, 21, 25, 21 and 48 kDa respectively. The expression of caspase-3 was detected as bands of molecular weight 35 kDa and 20 kDa and that of PARP was detected as a band of molecular weight 116 kDa and a cleaved product of molecular weight 85 kDa. The mean expression from control lysates was designated as 100% in the graph. Each bar represents the mean protein expression ± SD of 6 determinations per treatment. Topical application of DMBA significantly increased the expression of NF-κB, VEGF and Bcl-2 and decreased Bax, caspases -3, caspase-9, and PARP expression as well as caspase-3 activity compared to untreated control. Dietary administration of both P-B and BTF-35 to DMBA painted animals (groups 2 and 3) significantly decreased the expression of NF-κB, VEGF and Bcl-2 and increased the expression of Bax, caspase-3, caspase-9, PARP and its cleaved product as well as caspase-3 activity compared to group 1. However, modulation of protein expression and increase in caspase-3 activity was more significant in BTF-35 treated (group 3) animals compared to the P-B treated hamsters (group 2). In animals administered P-B and BTF-35 alone (groups 4 and 5), protein expression and the activity of caspase-3 were not significantly different as compared to untreated control.

## Discussion

In the present study, topical application of DMBA to the hamster cheek pouch for 14 weeks induced squamous cell carcinomas that displayed increased cell proliferation, infiltrative and angiogenic potential coupled with apoptosis evasion. Overexpression of cyclin D1, p21, GST-P, and NF-κB in HBP carcinomas is consistent with similar findings in DMBA-induced experimental tumours and in human OSCC reported in literature [17-21]. In particular, DMBA is known to induce the expression of GST-P and NF-κB [17,18]. Induction of GST-P is believed to facilitate cell proliferation and inhibit apoptosis leading to clonal expansion and malignant transformation [18]. p21, a critical downstream mediator of wild type p53 regulates several cell cycle proteins including cyclin D1, inhibits cyclin-dependent kinases (CDKs) and induces cell cycle arrest [22]. Although this implies downregulation of p21 expression in tumour cells, several reports including the present one have demonstrated overexpression of p21 in the cytoplasm of rapidly proliferating tumours [23,24]. These observations suggest that nuclear localization is essential for p21 to function as a CDK inhibitor. Upregulation of both p21 and cyclin D1 has been reported in several tumours including OSCCs [25,26]. In addition to cell cycle regulation, p21 also has a role in cell survival. Akita *et al *[27] demonstrated that p21^waf1 ^stimulates constitutive activation of NF-κB signaling in W_4 _cells independent of Rel A nuclear localization. Thus cytoplasmic colocalization and overexpression of p21 and NF-κB in HBP tumours may be interrelated and indicative of increased cell proliferation and cell survival.

In addition to their regulatory role in the cell cycle, GST-P, NF-κB and p21 also inhibit apoptosis by interacting with procaspase-3 and increasing the Bcl-2/Bax ratio [28]. Overexpression of Bcl-2 observed in HBP tumours in the present study can block mitochondrial mediated apoptosis by preventing the release of apoptogenic factors such as cytochrome C, thereby inhibiting activation of caspases–9 and -3, as well as cleavage of PARP, a nuclear enzyme involved in DNA repair and maintenance of genomic integrity. The antiapoptotic protein Bcl-2 is also recognized to act as a proangiogenic signaling molecule and potentiate NF-κB-mediated angiogenesis [29]. The proangiogenic factor VEGF in turn induces endothelial cell proliferation and Bcl-2 mediated cell survival [30]. Increased cell proliferation together with apoptosis evasion and neovascularization can facilitate tumour infiltration in HBP carcinomas as evidenced by overexpression of cytokeratins. The results of the present study demonstrate that cell proliferation, apoptosis, and angiogenesis are intricately interlinked in malignant transformation of the HBP mucosa by DMBA. Overexpression of Bcl-2, cytokeratins, and VEGF with downregulation of Bax, cytochrome C, caspase-3, caspase-9 and PARP may confer a survival advantage to HBP carcinomas by acquisition of an apoptosis-resistant, invasive and angiogenic phenotype.

Dietary administration of P-B and BTF-35 for 18 weeks starting from 4 weeks prior to DMBA exposure reduced the incidence of HBP carcinomas and preneoplastic lesions. The inhibitory effect of P-B and BTF-35 on HBP carcinogenesis was associated with enhanced nuclear expression of p21 and downregulation of NF-κB and GST-P expression. Studies have demonstrated that tea polyphenols exert their growth inhibitory effects on cancer cell lines by inducing p21 [31,32]. Nuclear localization of p21 induced by black tea polyphenols in the present study may facilitate cell cycle arrest by direct interaction with cyclin/CDK complex [33]. Although induction of p21 coincided with the impaired expression of cyclin D1, a study on CDK inhibitors suggests that p21 fails to inhibit cyclin D-CDK when bound to this complex at 1:1 ratio [34]. Therefore, in the present study p21 may decrease the expression of cyclin D1 through some indirect mechanism that needs to be elucidated. Consistent with our findings, Liberto and Cobrinik [35] demonstrated simultaneous induction of p21 expression with inhibition of cyclin D1-mediated pRB phosphorylation in MCF cells by EGCG. In addition to the effects on cell cycle regulators, tea has also been reported to downregulate NF-κB and GST-P in cell lines and animal tumour models [36,37]. Banerjee *et al *[38] found a positive correlation between the antiproliferative and proapoptotic effects of black tea polyphenols during benzo [a]pyrene-induced lung carcinogenesis. The decrease in Bcl-2/Bax ratio, a reliable indicator of the overall propensity of a cell to undergo apoptosis coupled with overexpression of cytochrome C, caspases-9, -3 and cleavage of PARP by P-B and BTF-35 in the present study strengthens the apoptosis inducing potential of black tea polyphenols reported in cell lines and animal models by other workers [39-42].

The antiproliferative and apoptosis inducing effects of P-B and BTF-35 seen in the present study could potentially mitigate the carcinogenic effects of DMBA thereby decreasing the infiltrative and angiogenic potential of HBP carcinomas as reflected by decreased cytokeratin and VEGF expression. A previous study from this laboratory demonstrated a positive association between downregulation of cytokeratin AE1/AE3 expression and inhibition of HBP carcinogenesis by lactoferrin and black tea polyphenol combination [10]. Tea polyphenols have been reported to inhibit angiogenesis by downregulating VEGF expression [43].

## Conclusion

The results of the present study provide evidence that P-B and BTF-35 act as suppressing agents by inhibiting cell proliferation, tumour cell survival, infiltration and angiogenesis, and inducing apoptosis. An interesting finding in the present study is the differential sensitivities of HBP carcinomas and normal pouch to growth control and apoptosis induction by P-B and BTF-35. Administration of P-B and BTF-35 selectively induced apoptosis in DMBA painted hamsters but not in normal animals. Tea polyphenols such as EGCG and theaflavins have been demonstrated to induce apoptosis of tumour cells while sparing normal cells [44,45]. Taken together, these findings potentiate the fact that diet-derived agents such as P-B and BTF-35 that drive tumour cells to undergo apoptosis but direct normal cells towards a survival pathway are ideal chemopreventives.

The greater efficacy of BTF-35 may be attributed to the higher content of theaflavins and low caffeine. Theaflavins have been reported to act on multiple signal transduction pathways resulting in induction of apoptosis and suppression of NF-κB and COX-2 expression [41]. Low dose caffeine has been reported to be more beneficial than higher doses in suppressing cell proliferation and inducing apoptosis in cancer cell lines [46,47]. The results of the present study suggest that BTF-35 may have an immense potential in human oral cancer prevention. However, additional studies on the effect of BTF-35 on a wide range of cell cycle associated proteins, NF-κB signaling pathways, Bcl-2 family proteins, caspases, and angiogenic chemokines are required to unravel the differential response of BTF-35 in normal versus cancer cells and confirm the mechanistic pathways of chemoprevention.

## Abbreviations

CDK – cyclin-dependent kinase

DMBA – 7,12-dimethylbenz [a]anthracene

GST-P – glutathione S-transferase-pi

HBP – hamster buccal pouch

NF-κB – nuclear factor kappa B

OSCC – oral squamous cell carcinoma

PARP – poly(ADP-ribose)polymerase

P-B – Polyphenon-B

VEGF – vascular endothelial growth factor

## Competing interests

The author(s) declare that they have no competing interests.

## Authors' contributions

SN is a researcher working in cancer biology and she designed the overall study. PV and CM undertook the RT-PCR, immunohistochemical and Western blot analyses. DP designed the immunohistochemical analysis and interpreted the results. YH fractionated and analysed the black tea polyphenols using HPLC. PV and CM contributed to the writing of the manuscript, which was edited, revised critically and co-ordinated by SN. All the authors read and approved the final manuscript.
